# Geophagy During Pregnancy in Sub‐Saharan Africa: A Review of Cultural Practices and Health Implications

**DOI:** 10.1155/sci5/9219293

**Published:** 2026-10-25

**Authors:** Kelvin E. Vulla, Margaret Maoni, Victoria Ndolo

**Affiliations:** ^1^ Department of Chemistry and Chemical Engineering University of Malawi Zomba Malawi unima.mw; ^2^ Center for Resilient Agri-Food Systems (CRAFS) University of Malawi Zomba Malawi unima.mw; ^3^ Department of Food Science and Technology (DFST) University of Dar es Salaam (UDSM) Dar es Salaam Tanzania udsm.ac.tz; ^4^ Department of Human Ecology and Agricultural Sciences University of Malawi Zomba Malawi unima.mw

**Keywords:** geophagy, iron deficiency anaemia, maternal nutrition, pregnancy, sub-Saharan Africa

## Abstract

Geophagy, the intentional consumption of soil and other earth materials, is a common practice among pregnant women in many parts of sub‐Saharan Africa. This narrative review aimed to examine the cultural drivers, nutritional implications and health consequences of geophagy during pregnancy, as well as identify potential intervention strategies for mitigating associated risks. Literature retrieved from PubMed, Scopus, Web of Science, ScienceDirect and Google Scholar was synthesized to examine evidence on geophagy during pregnancy in sub‐Saharan Africa. The available evidence indicates that geophagy during pregnancy is influenced by a combination of cultural traditions, social norms, physiological changes and perceived health benefits. While the practice is often associated with attempts to alleviate pregnancy‐related symptoms and address nutritional deficiencies, it may contribute to adverse maternal and foetal outcomes through reduced nutrient bioavailability, exposure to toxic elements and increased susceptibility to parasitic infections. Evidence from the reviewed studies also indicates associations between geophagy and maternal anaemia, neural tube defects, low birth weight and preterm birth. Despite these risks, routine screening and culturally appropriate counselling regarding geophagy remain inadequately incorporated into antenatal care services across many settings in sub‐Saharan Africa. This review highlights the need for culturally sensitive public health interventions, improved maternal nutrition education and strengthened research efforts to better understand the determinants and health implications of geophagy during pregnancy. Integrating geophagy‐related screening and counselling into maternal healthcare programmes may contribute to improved pregnancy outcomes and maternal well‐being in the region.

## 1. Introduction

Maternal anaemia and neural tube defects (NTDs) continue to pose significant public health challenges worldwide, particularly in low‐ and middle‐income countries. Anaemia is one of the most common nutritional and health problems affecting pregnant women and young children, with the greatest burden occurring in low‐resource settings. Recent global estimates indicate that approximately 37% of pregnant women are affected by anaemia, while children under 5 years also experience a substantial burden of anaemia, particularly in the WHO African Region [1]. Anaemia during pregnancy is associated with increased risks of maternal morbidity and mortality, preterm birth, low birth weight and impaired development of offspring [2, 3]. Similarly, NTDs, including spina bifida and anencephaly, remain important congenital anomalies that contribute to neonatal mortality and lifelong disability [4, 5]. Adequate maternal nutrition, particularly sufficient intake and bioavailability of iron and folate, therefore plays a critical role in supporting maternal health and foetal development.

Iron and folate are essential micronutrients during pregnancy because they support maternal physiological adaptation and foetal development. Iron is required for haemoglobin synthesis, red blood cell production and oxygen transport, while the increased blood volume and iron requirements of pregnancy increase the risk of iron deficiency and anaemia when dietary intake and stores are inadequate [6]. Folate is essential for DNA synthesis, cell division and neural tube formation during early embryonic development [7]. Deficiencies in these nutrients have been associated with adverse pregnancy outcomes, including maternal anaemia, NTDs, impaired foetal growth and preterm birth [4, 8]. Although iron and folic acid supplementation remain central components of maternal health programmes, micronutrient deficiencies continue to persist in many populations because of inadequate dietary intake, limited nutrient bioavailability, infections and behavioural practices that may interfere with nutrient absorption.

Among the culturally embedded practices observed during pregnancy, geophagy, the intentional consumption of soil, clay, or other earth materials has received particular attention because of its potential nutritional and health implications for mothers and their offspring [9, 10]. Geophagy is a specific form of pica and has been documented across different cultures and historical periods, with evidence of the practice extending to ancient societies. In Africa, it has been reported among different population groups, but pregnant women and children are among the groups in whom the practice is most frequently documented [11]. Studies have reported substantial variation in the prevalence of geophagy during pregnancy across African settings, reflecting differences in cultural practices, geographical location, study populations and methods of assessment (Figure 1) [12]. Pregnancy may provide a particularly important context for geophagy because physiological and nutritional changes during this period can be accompanied by nausea, gastrointestinal discomfort, food cravings and altered appetite. In addition, geophagy may be culturally accepted and perceived by pregnant women as providing medicinal, nutritional, protective, or symptomatic benefits [12]. The persistence of the practice despite increasing access to biomedical antenatal care highlights the importance of understanding geophagy not only as an individual behaviour but also as a culturally embedded practice with potential implications for maternal and child health.

**FIGURE 1:**
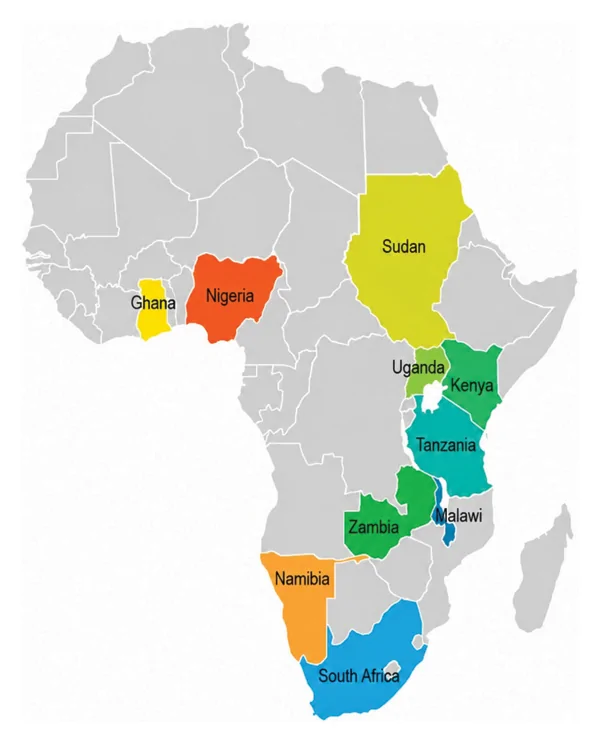
Countries in sub‐Saharan Africa with documented geophagy practices among pregnant women [12].

Of particular concern is the potential relationship between geophagy, maternal micronutrient status and anaemia. Geophagic materials may contain minerals, but their nutritional contribution can vary considerably according to their geological and mineralogical composition, and some materials may interfere with the absorption and bioavailability of dietary micronutrients through adsorption, binding, or other physicochemical interactions [13]. Evidence from Tanzania, for example, has reported associations between geophagy and anaemia‐related haematological indicators among pregnant women, while also identifying an association between geophagy and *Ascaris lumbricoides* infection [14]. However, the evidence is not entirely consistent, and the relationship may be influenced by other determinants of maternal anaemia, including dietary intake, infection, supplementation and socioeconomic factors. Geophagic materials may also expose pregnant women to potentially toxic elements, environmental contaminants and parasitic organisms, creating additional concerns for maternal health and foetal development [15, 16]. These potential pathways are important because maternal nutritional deficiencies, infections and toxic exposures during pregnancy may have consequences extending beyond the mother to the developing foetus and, subsequently, child health.

The importance of studying geophagy during pregnancy therefore extends beyond the practice itself to its potential interaction with major maternal and child health challenges, particularly anaemia and adverse pregnancy outcomes. Pregnant women are already vulnerable to micronutrient deficiencies because of increased physiological demands, while the developing foetus depends on maternal nutrient availability for growth and development. Consequently, behaviours that may reduce nutrient bioavailability or increase exposure to contaminants warrant careful investigation. At the same time, geophagy may provide perceived or culturally valued benefits to women, meaning that interventions based solely on discouraging the practice may be ineffective if they do not address the underlying cultural, physiological and nutritional motivations.

Although numerous studies have investigated geophagy in Africa, the evidence remains fragmented across cultural, nutritional, toxicological, infectious and maternal health perspectives. Previous reviews have highlighted substantial variation in prevalence, proposed motivations and health effects, while also identifying continuing gaps in understanding the biological mechanisms and health consequences of geophagy during pregnancy [11, 12]. In particular, the relationship between geophagy and maternal anaemia remains incompletely characterized and evidence linking the practice with foetal and subsequent child health outcomes is comparatively limited. Furthermore, geophagy is not consistently incorporated into antenatal screening, nutrition counselling, or maternal health interventions in many African settings. A comprehensive synthesis that brings together the cultural drivers, nutritional implications, potential toxicological and infectious risks and maternal and foetal health consequences is therefore warranted.

Therefore, this narrative review aims to synthesize current evidence on the cultural practices, nutritional consequences and health implications of geophagy during pregnancy in sub‐Saharan Africa, with particular emphasis on its relationship with maternal anaemia and adverse pregnancy outcomes, while identifying knowledge gaps and priorities for future research and intervention.

## 2. Methodology

### 2.1. Review Design

This study was conducted as a narrative review to synthesize existing evidence on geophagy during pregnancy in sub‐Saharan Africa, with particular emphasis on cultural practices, nutritional consequences and maternal and foetal health implications. Relevant literature was identified through searches of major scientific databases, including PubMed, Scopus, Web of Science, ScienceDirect and Google Scholar. Additional relevant publications were identified through manual screening of reference lists of selected articles.

### 2.2. Search Strategy

The literature search employed combinations of keywords and Boolean operators related to geophagy, pregnancy, maternal nutrition, anaemia and health outcomes. The main search terms included “geophagy,” “pica,” “pregnancy,” “pregnant women,” “soil consumption,” “clay consumption,” “maternal nutrition,” “iron deficiency anaemia,” “neural tube defects,” “health outcomes,” and “Sub‐Saharan Africa.” The Boolean operators “AND” and “OR” were used to combine the search terms. Examples of search combinations included (“geophagy” OR “pica” OR “soil consumption” OR “clay consumption”) AND (“pregnancy” OR “pregnant women”) AND (“anaemia” OR “iron deficiency” OR “maternal health”) AND (“Sub‐Saharan Africa” OR “Africa”). Additional relevant publications were identified through manual screening of reference lists from selected articles.

### 2.3. Inclusion Criteria

Literature was included if it examined geophagy or pica during pregnancy and reported information relevant to its cultural, nutritional, toxicological, or health implications. Studies focussing on pregnant women and maternal health outcomes were prioritized. Publications conducted in sub‐Saharan Africa were preferentially considered, although studies from other regions were included where they provided relevant contextual or comparative information.

Eligible sources included peer‐reviewed journal articles, review papers, books, conference proceedings and reports from recognized national and international organizations. Only publications written in English were considered.

### 2.4. Exclusion Criteria

Publications were excluded if they did not address geophagy or pica during pregnancy, focused exclusively on nonpregnant populations without relevance to maternal health, or lacked sufficient information relevant to the objectives of the review. Duplicate publications were not considered more than once. Editorials, opinion articles, commentaries, news reports and other nonscholarly publications without supporting evidence were also excluded. Publications written in languages other than English were not considered.

### 2.5. Narrative Synthesis

The identified literature was examined in relation to the main objectives of the review and synthesized narratively. The evidence was organized around the major themes addressed in the review, including the occurrence and cultural context of geophagy during pregnancy, perceived reasons for its practice, nutritional implications, potential health risks, associations with maternal anaemia and other pregnancy outcomes and potential strategies for addressing geophagy within maternal healthcare. Relevant findings from individual publications were integrated thematically to provide an overall understanding of geophagy during pregnancy in sub‐Saharan Africa. As this was a narrative review, no quantitative meta‐analysis or statistical pooling of findings was performed.

### 2.6. Reporting Considerations

The reporting of this narrative review was guided by the Scale for the Assessment of Narrative Review Articles (SANRA), a framework developed specifically to improve the quality and transparency of narrative reviews [17].

## 3. Epidemiology of Geophagy

### 3.1. Prevalence Globally and in SSA

The prevalence of geophagy during pregnancy (GiP) varies considerably both between and within countries. Globally, approximately 36% of pregnant women are estimated to engage in geophagy, with prevalence rates reaching as high as 73% in some populations of sub‐Saharan Africa [1]. Studies from several countries in the region indicate that geophagy remains a common practice during pregnancy, often influenced by cultural beliefs, traditional norms and perceived health benefits, including the alleviation of pregnancy‐related discomforts [1]. As illustrated in Figure 2, prevalence estimates differ substantially across countries and populations.

**FIGURE 2:**
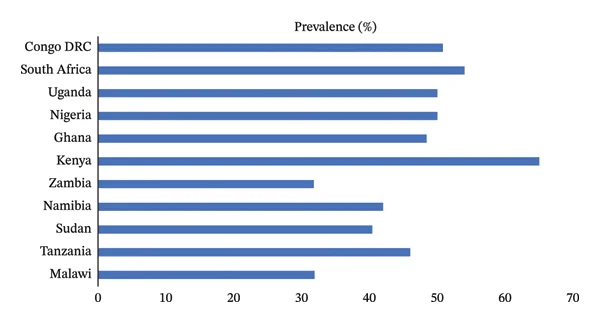
Reported prevalence of geophagy among pregnant women in selected sub‐Saharan African countries.

For example, in Malawi, approximately 31.9% of pregnant women reportedly consume soil, with higher prevalence observed in rural communities where cultural acceptance tends to be stronger than in urban settings [16]. Similar practices have been documented in other sub‐Saharan African countries, highlighting the widespread nature of geophagy during pregnancy across the region [18–25].

### 3.2. Variation by Pregnancy Status, Trimester and Region

#### 3.2.1. Pregnancy Status

Geophagy is often more prevalent among pregnant women than nonpregnant women, driven by cravings, hormonal changes, or perceived health benefits [26]. For example, in Nigeria, studies showed that 50% of pregnant women practiced geophagy compared to 10% of nonpregnant women [27].

#### 3.2.2. Trimester

Cravings for earth substances tend to peak during the second and third trimesters, coinciding with increased nutritional demands and hormonal fluctuations. A study in Ghana reported that 60% of pregnant women in their second trimester engaged in geophagy, compared to 30% in the first trimester [21].

#### 3.2.3. Regional Variations

Geography influences prevalence due to cultural beliefs, availability of earth materials and socioeconomic factors. For example, in West Africa, high prevalence linked to traditional medicinal practices and beliefs about soil’s medicinal properties [28]. In East Africa, slightly lower rates, but still significant, often associated with pregnancy cravings [23, 29]. In Southern Africa, lower prevalence, possibly due to differing cultural norms and increased health education [30]. In Nigeria’s northern regions [22], prevalence can reach up to 50%, whereas in urban South Africa [30], it may be less than 10%, reflecting differences in cultural acceptance and awareness.

### 3.3. Common Types of Geophagic Materials

The materials consumed through geophagy vary significantly, depending on factors such as local geology, cultural preferences and the availability of specific substances. These commonly include various types of soils and clays, some of which are illustrated in Figure 3. The choice of material is often influenced by traditional practices and the perceived health benefits associated with specific earth substances. Common types include:

**FIGURE 3:**
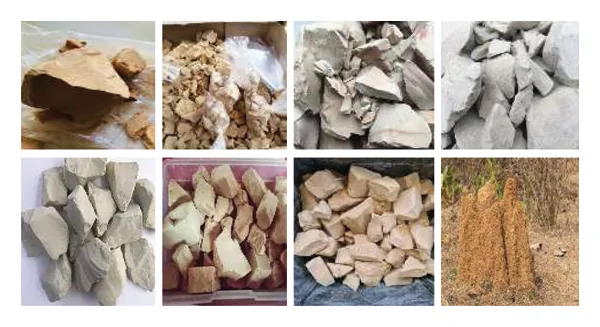
Common geophagic materials consumed by pregnant women in different regions of sub‐Saharan Africa.

#### 3.3.1. Clay‐Rich Soils

Clay‐rich soils, such as bentonite, kaolin and other fine clay minerals, are among the most commonly consumed geophagic substances [31]. These clays are believed to have soothing properties, alleviating nausea and gastrointestinal discomfort during pregnancy, while also providing essential minerals like calcium and magnesium [32]. In Nigeria, women frequently consume a type of clay known as ‘pica clay’ or ‘pottery clay’, which they believe helps reduce morning sickness and supplies vital nutrients, reinforcing the cultural association between clay ingestion and health benefits [33].

#### 3.3.2. Fertile Earths

Fertile, loamy soils rich in organic matter are also consumed in various regions. These soils are thought by some cultures to improve overall health, increase energy, or promote vitality. For example, in parts of Ethiopia, pregnant women often eat earth from highly fertile soils, believing it enhances strength and sustains them during pregnancy [34]. The organic content and mineral richness of these soils reinforce their perceived health‐boosting properties.

#### 3.3.3. Mud or Dirt From Specific Sites

Certain geophagic practices involve soil collected from specific natural sites such as riverbanks, termite mounds, or areas near trees [35]. These soils are often linked with spiritual or medicinal beliefs. In Kenya, women frequently chew or swallow soil from riverbanks, which is believed to contain high mineral content and is associated with fertility and vitality [36]. Similarly, in Ethiopia, earth from termite mounds is popular, as it is considered to be rich in nitrogen and minerals that boost energy levels and general health [37]. The cultural significance of these sites often ties the soil to spiritual well‐being and traditional healing practices.

#### 3.3.4. Specific Mineral‐Rich Soils

Some geophagic materials are distinguished by their high content of particular minerals, such as calcium, magnesium, or trace elements, which are thought to address nutritional deficiencies. For instance, certain soils are consumed because they are rich in essential minerals, with the belief that they help prevent deficiencies and support foetal development [38]. These mineral‐rich soils are often selected intentionally based on their mineral profile, further highlighting the cultural and nutritional significance attached to specific earth materials.

### 3.4. Conceptual Framework

The conceptual framework presented in Figure 4 illustrates the interrelationships among the key determinants of geophagy during pregnancy and their potential implications for maternal nutrition and pregnancy outcomes. The framework proposes that sociocultural, biological, nutritional and health‐system factors influence geophagic practices, which may subsequently affect maternal nutritional status and pregnancy outcomes through various nutritional and toxicological pathways.

**FIGURE 4:**
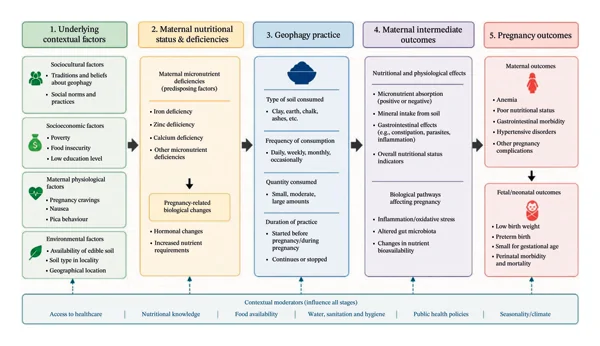
Conceptual framework of the determinants, pathways and health implications of geophagy during pregnancy.

### 3.5. Determinants of Geophagy During Pregnancy

#### 3.5.1. Cultural and Social Influences

Cultural beliefs and social norms play a central role in shaping geophagy practices among pregnant women. In many societies, consuming earth substances is deeply rooted in tradition and is often associated with health, fertility and spiritual well‐being. For example, in Nigeria and Ghana, geophagy has been linked to cultural perceptions of womanhood, fertility and maternal strength [21, 22]. These beliefs are frequently reinforced through family traditions, community narratives, rituals and advice from elders, making geophagy a socially accepted or even expected behaviour during pregnancy [39]. In some communities, refusing to consume earth materials may be perceived as disregarding cultural expectations or increasing the risk of adverse pregnancy outcomes. Consequently, cultural acceptance contributes significantly to the persistence and intergenerational transmission of geophagic practices.

#### 3.5.2. Nutritional and Physiological Factors

Nutritional deficiencies and physiological changes during pregnancy are among the most frequently cited factors associated with geophagy. Deficiencies in iron, zinc and other micronutrients have long been proposed as potential drivers of soil consumption, particularly in settings where dietary diversity is limited [28, 40, 41]. Pregnancy also involves substantial physiological changes, including hormonal fluctuations, altered taste perception, nausea, vomiting and gastrointestinal discomfort, which may contribute to cravings for nonfood substances, including soil [41–43]. Consequently, many pregnant women report engaging in geophagy as a means of alleviating morning sickness or managing digestive discomfort [16].

Although some women perceive geophagic materials as sources of essential minerals, scientific evidence regarding this hypothesis remains inconclusive. While certain soils contain measurable amounts of minerals, studies suggest that many geophagic materials may reduce the bioavailability of micronutrients through adsorption, chelation and mineral‐binding mechanisms, thereby potentially exacerbating existing nutritional deficiencies rather than correcting them [13]. For example, the consumption of termite mound soil has been reported among pregnant women in Namibia, where the practice is often associated with perceived nutritional benefits and traditional beliefs regarding maternal health and foetal development [12]. These findings suggest that physiological responses to pregnancy and perceived nutritional needs may interact to influence geophagic behaviour.

#### 3.5.3. Behavioural and Psychological Factors

Behavioural and psychological factors may further influence the initiation and continuation of geophagy during pregnancy. Some women develop habitual patterns of soil consumption following repeated exposure during previous pregnancies or through family and community practices [44]. In addition, psychological factors such as stress, anxiety and emotional distress have been associated with increased likelihood of engaging in geophagic behaviour [45]. For example, studies from Nigeria suggest that some pregnant women may use soil consumption as a coping mechanism for stress or emotional discomfort during pregnancy [22]. These behavioural influences often interact with cultural expectations and physiological cravings, reinforcing the persistence of geophagy throughout pregnancy.

#### 3.5.4. Health System and Counselling Factors

The persistence of geophagy is also influenced by gaps within healthcare systems, particularly inadequate antenatal education, nutritional counselling and behavioural support. Many pregnant women remain unaware of the potential health risks associated with soil consumption, while healthcare providers may lack sufficient training or resources to identify and address geophagic practices effectively [11]. For example, women in rural South Africa have reported limited access to information regarding the potential consequences of geophagy and often rely on traditional beliefs and community advice instead [46]. The absence of routine screening and culturally sensitive counselling during antenatal care may contribute to the continuation of geophagy during pregnancy. Strengthening nutrition education, risk communication and behavioural counselling within maternal healthcare services could therefore play an important role in reducing geophagy and promoting safer alternatives for managing pregnancy‐related symptoms.

### 3.6. Nutritional and Biological Effects of Geophagy

#### 3.6.1. Effects on Nutrient Bioavailability and Micronutrient Status

One of the principal concerns associated with geophagy during pregnancy is its potential to affect the bioavailability of essential nutrients, particularly iron, folate and zinc. Many geophagic materials contain clay minerals and other compounds capable of adsorbing micronutrients within the gastrointestinal tract, thereby reducing their absorption and utilization [47–49]. Studies conducted in several sub‐Saharan African settings have reported associations between geophagy and lower iron status indicators, including reduced serum ferritin concentrations and increased prevalence of anaemia among pregnant women [47, 48]. In addition, soil minerals such as calcium may compete with iron for absorption pathways, potentially further limiting micronutrient availability [25].

These interactions may also reduce the effectiveness of iron–folic acid supplementation programmes when soil consumption occurs concurrently with supplement use [14, 22]. Although some geophagic materials contain measurable amounts of minerals, current evidence generally suggests that the inhibitory effects of soil on nutrient bioavailability may outweigh any potential nutritional contribution. Nevertheless, the magnitude of these effects appears to vary according to the composition, quantity and frequency of soil consumption [13, 48, 49]. Further research is therefore needed to clarify the extent to which geophagy influences micronutrient status during pregnancy.

#### 3.6.2. Toxicological and Gastrointestinal Effects

In addition to its nutritional implications, geophagy may expose pregnant women to toxicological and gastrointestinal risks. Geophagic materials can contain potentially harmful substances, including lead, arsenic, cadmium and mercury, which may accumulate in the body following repeated exposure [13, 14]. Studies from Ghana and Nigeria have reported elevated concentrations of heavy metals in soils commonly consumed by pregnant women, raising concerns regarding maternal and foetal exposure [21, 50]. Furthermore, contaminated soils may harbour parasitic organisms and pathogenic microorganisms capable of causing gastrointestinal infections and other adverse health effects [51].

The physical characteristics of some soils may also contribute to gastrointestinal complications, particularly when consumed frequently or in large quantities. Reported effects include constipation, abdominal discomfort and, in rare cases, gastrointestinal obstruction or impaction [52]. These findings suggest that geophagy may present multiple biological risks during pregnancy, though the severity of these effects is likely influenced by the type, composition and quantity of soil consumed.

### 3.7. Geophagy and Maternal Health Outcomes

Evidence from several studies suggests that geophagy may be associated with adverse maternal health outcomes, particularly through its effects on micronutrient status and gastrointestinal health. Among the most consistently reported associations is the relationship between geophagy and iron deficiency anaemia. Geophagic materials often contain clay minerals and other compounds capable of binding iron within the gastrointestinal tract, potentially reducing its bioavailability and contributing to poor iron status [25, 53–55]. Consequently, pregnant women who practice geophagy have frequently been reported to exhibit lower serum ferritin concentrations and higher prevalence of anaemia than nongeophagic women [25, 54, 55]. However, because most available studies are observational, the direction and magnitude of this relationship remain difficult to establish conclusively.

Beyond iron deficiency, geophagy has also been associated with altered status of other micronutrients, including zinc, calcium and folate [21, 56, 57]. Reduced bioavailability of these nutrients may adversely affect maternal nutritional status and physiological function, though the extent of these effects appears to vary according to the type and quantity of soil consumed [13, 57]. In addition, geophagy has been linked to gastrointestinal disturbances such as constipation, abdominal discomfort and, in rare cases, intestinal obstruction, particularly when coarse soil particles are consumed frequently [13, 58, 59].

Potential exposure to toxic elements represents another important maternal health concern. Several studies have identified elevated concentrations of heavy metals, including lead, arsenic and cadmium, in geophagic materials consumed by pregnant women [14, 15, 21, 50]. Chronic exposure to these contaminants may increase the risk of adverse maternal and foetal health effects, though exposure levels vary substantially across geographical locations and soil sources. Collectively, current evidence suggests that geophagy may contribute to nutritional, gastrointestinal and toxicological challenges during pregnancy, but further longitudinal studies are needed to clarify causal relationships and quantify associated risks.

### 3.8. Implications for Pregnancy Outcomes

The potential implications of geophagy for pregnancy outcomes are thought to arise primarily through its associations with maternal nutritional deficiencies, anaemia and exposure to environmental contaminants. Several studies have reported associations between geophagy and adverse pregnancy outcomes, including low birth weight, preterm delivery and impaired foetal growth [12, 22, 53, 57]. These outcomes are often hypothesized to result from reduced nutrient bioavailability and compromised maternal nutritional status rather than from direct effects of soil consumption itself.

Particular attention has been given to the possible relationship between geophagy, folate status and NTDs. Because folate plays a critical role in early neural tube development, any factor that interferes with folate intake or absorption may theoretically increase NTD risk [60]. However, direct evidence linking geophagy to NTDs remains limited, and currently available studies are insufficient to establish a causal relationship. Consequently, the proposed association should be interpreted cautiously and requires further investigation [57, 60, 61].

Geophagy may also indirectly influence pregnancy outcomes through its association with maternal anaemia and poor nutritional status. Maternal anaemia has been linked to increased risks of low birth weight, preterm birth, impaired foetal growth and adverse maternal outcomes [62, 63]. Therefore, if geophagy contributes to micronutrient deficiencies or worsens existing nutritional challenges, it may indirectly increase susceptibility to these complications. Nevertheless, the complexity of nutritional, socioeconomic and healthcare‐related factors influencing pregnancy outcomes makes it difficult to isolate the specific contribution of geophagy from other coexisting determinants.

### 3.9. Perceived and Potential Benefits of Geophagy

Despite the documented concerns associated with geophagy, several hypotheses have been proposed to explain its persistence among pregnant women. One commonly cited explanation is the perception that certain soils provide minerals or other nutritional benefits, particularly in settings where dietary diversity is limited [12, 64]. Although some geophagic materials contain measurable quantities of minerals, current evidence does not consistently demonstrate that these minerals are sufficiently bioavailable to improve nutritional status. The nutritional benefit hypothesis remains inconclusive.

Another proposed explanation relates to the adsorptive properties of some clay minerals, particularly kaolin and bentonite. These materials have historically been used in traditional medicine and pharmaceutical preparations because of their ability to bind certain substances within the gastrointestinal tract [65–67]. This property has led to suggestions that geophagic materials may help alleviate gastrointestinal discomfort or reduce exposure to certain toxins. However, evidence supporting these effects in pregnant women practicing geophagy remains limited and should be interpreted cautiously.

Many women also report consuming soil to alleviate pregnancy‐related symptoms such as nausea, vomiting and heartburn [19, 68]. While these perceived benefits may help explain the cultural persistence of geophagy, they do not eliminate the potential nutritional and toxicological risks associated with the practice [15]. Overall, the available evidence suggests that the perceived benefits of geophagy are largely based on cultural beliefs, personal experiences and biological hypotheses rather than well‐established clinical evidence. Further research is needed to determine whether any specific geophagic materials possess measurable health benefits that outweigh their potential risks.

## 4. Toxicological Implications of Geophagy: Heavy Metals and Carcinogenic Risks

### 4.1. Heavy Metal Contamination and Exposure Pathways

Geophagic materials consumed by pregnant women in sub‐Saharan Africa frequently contain potentially toxic heavy metals, including lead (Pb), arsenic (As), mercury (Hg), cadmium (Cd) and chromium (Cr), originating from both natural geological formations and environmental contamination [69, 70]. Studies conducted in Tanzania, Kenya, Ghana, Nigeria and other parts of the region have reported elevated concentrations of these metals in geophagic materials sold in local markets and commonly consumed during pregnancy [9, 12, 29, 69]. In several instances, detected concentrations exceeded internationally recommended safety limits, suggesting that geophagy may represent an important route of exposure to environmental contaminants. Common heavy metals identified in geophagic materials and their associated health risks are presented in Table 1.

**TABLE 1:** Common heavy metals identified in geophagic materials and their associated health risks.

Heavy metal	Common presence	Major health risk
Lead (Pb)	Very common	Neurotoxicity, anaemia and cancer risk
Arsenic (As)	Natural geological deposits	Carcinogen (skin, lung and bladder cancers)
Cadmium (Cd)	Agricultural and environmental contamination	Kidney damage and cancer
Mercury (Hg)	Industrial contamination	Neurotoxicity, developmental toxicity
Chromium (Cr)	Industrial areas	Strong carcinogen

Once ingested, heavy metals may become bioavailable within the acidic environment of the gastrointestinal tract and subsequently enter systemic circulation [71]. During pregnancy, physiological adaptations that enhance nutrient absorption to support foetal growth may also increase the absorption of certain toxic metals. Following absorption, these contaminants can accumulate in maternal tissues and cross the placental barrier, exposing the developing foetus during critical stages of growth and development [72]. Such exposure is of particular concern because foetal detoxification systems are not fully developed, potentially increasing vulnerability to toxic effects.

### 4.2. Potential Health and Carcinogenic Risks

Exposure to heavy metals through geophagy may contribute to a range of adverse health outcomes. Several metals commonly detected in geophagic materials have been classified as carcinogenic or potentially carcinogenic to humans. Arsenic and cadmium are classified by the International Agency for Research on Cancer (IARC) as Group 1 carcinogens, while lead is classified as a probable human carcinogen [73]. These substances have been associated with biological mechanisms including oxidative stress, DNA damage, genomic instability and disruption of normal cellular regulatory processes [74, 75].

Pregnancy represents a particularly sensitive period for environmental exposures. According to the Developmental Origins of Health and Disease (DOHaD) framework, exposure to environmental toxicants during prenatal development may influence health outcomes later in life [76, 77]. Consequently, prenatal exposure to heavy metals through geophagy has raised concerns regarding both immediate and long‐term health consequences for mothers and their offspring. However, while biological plausibility exists, the extent to which geophagy‐related heavy metal exposure contributes to future carcinogenic outcomes remains insufficiently characterized and requires further investigation.

In addition to carcinogenic concerns, heavy metal exposure may interfere with nutrient metabolism and utilization. Lead and cadmium, for example, have been reported to disrupt the absorption and metabolism of essential micronutrients, potentially exacerbating nutritional deficiencies and anaemia among vulnerable populations [78, 79].

### 4.3. Evidence From Sub‐Saharan Africa and Public Health Implications

Evidence from sub‐Saharan Africa highlights the widespread occurrence of heavy metal contamination in geophagic materials and underscores the potential public health implications of this practice. Although geophagy is often practiced to alleviate pregnancy‐related symptoms or address perceived nutritional needs, the consumption of contaminated soils may unintentionally increase exposure to toxic substances [9, 12, 29, 69]. Given the coexistence of geophagy, micronutrient deficiencies and limited environmental monitoring in many settings, understanding these exposure pathways remains an important public health priority.

Addressing the toxicological risks associated with geophagy requires a multifaceted approach that combines maternal nutrition interventions, risk communication and culturally sensitive health education. Integrating geophagy screening and counselling into routine antenatal care may help improve awareness of potential risks while respecting the cultural contexts in which the practice occurs. Table 2 summarizes selected studies from sub‐Saharan Africa that have investigated heavy metal contamination in geophagic materials and associated health risks.

**TABLE 2:** Summary of studies from sub‐Saharan Africa reporting heavy metal contamination in geophagic materials and associated health risks.

Author (s)	Study location	Sample type	Analysed metals	Carcinogenic or health risk found	Key comments
Nkansah et al. [80]	Kumasi, Ghana	White clay	Arsenic (As), lead (Pb), mercury (Hg), cadmium (Cd), calcium (Ca), iron (Fe), potassium (K), zinc (Zn)	The results indicated that consumers are likely to suffer from cancer through ingestion of geophagic clay, especially due to high levels of arsenic and lead	The clay samples contained both essential elements and toxic metals like As and Pb. Certain heavy metal levels exceeded WHO/FAO’s Permitted Maximum Tolerable Daily Intake (PMTDI), posing potential health threats over time
Mouri et al. [81]	Limpopo Province, South Africa	Twelve geophagic material samples	Arsenic (As), chromium (Cr), lead (Pb), along with other major and trace elements (via XRF and ICP‐MS)	Higher than recommended daily intake levels of nonessential elements like arsenic, chromium and lead, indicating potential health risks	Based on geochemical content and risk assessment, the study concludes that these geophagic materials are unsuitable for human consumption
Kortei et al. [82]	Anfoega, Volta region, Ghana	Clay samples used as pica by pregnant women	Arsenic (As), manganese (Mn), lead (Pb), nickel (Ni), iron (Fe), copper (Cu), zinc (Zn), potassium (K), magnesium (Mg), sodium (Na)	The Target Hazard Quotients (THQ) values for arsenic, lead and nickel exceeded 1, suggesting potential health risks over a lifetime. The overall cumulative risk (TTHQ) was exceedingly high (≤ 15), implying significant safety concerns associated with consuming this clay	The high THQ for arsenic, lead and nickel underscores potential detrimental effects on health, especially during pregnancy. The cumulative toxic effects suggest that consuming this clay as pica is unsafe for pregnant women, as it may adversely affect the foetus
Ekosse et al. [83]	Markets in Cameroon (specific markets not named)	Geophagic clay	Nickel (Ni), chromium (Cr), zinc (Zn), cobalt (Co), iron (Fe), copper (Cu), lead (Pb)	The noncarcinogenic risk indices (for Fe, Co, Cr, Cu, Ni, Pb, Zn) were generally below 1, indicating no significant noncarcinogenic health risk from these metals for both children and adults. The carcinogenic risk index (TCR) for nickel and chromium was above 10^−6^, suggesting a minimal but present carcinogenic risk	The study emphasizes the potential health risks associated with consuming these geophagic clays, especially regarding nickel and chromium’s carcinogenic potential
Rukondo et al. [84]	Open markets in Morogoro, Njombe and Mwanza regions, Tanzania	Dry soil sticks consumed by women	Iron (Fe), calcium (Ca), potassium (K), zinc (Zn), sodium (Na), copper (Cu), manganese (Mn), lead (Pb), nickel (Ni), chromium (Cr), cadmium (Cd)	Prolonged exposure to lead, even at low concentrations, can cause pathological health effects. The presence of essential minerals alongside toxic metals poses health risks, especially with continued consumption	The findings suggest that, due to the presence of toxic metals with carcinogenic potential, prolonged geophagy should be discouraged to prevent serious health effects

## 5. Intervention Strategies and Programmatic Responses

Current evidence suggests that addressing geophagy during pregnancy requires interventions that are both evidence‐based and culturally responsive. Despite the potential nutritional and toxicological risks associated with geophagy, routine antenatal care (ANC) services in many low‐ and middle‐income settings do not consistently screen for soil consumption practices or provide counselling on their potential health implications [11, 46]. Consequently, opportunities to identify women practicing geophagy and offer appropriate nutritional guidance may be missed. In addition, concerns about stigma or disapproval may discourage some women from disclosing geophagic practices during ANC consultations, further limiting opportunities for intervention [46].

The cultural significance of geophagy presents an additional challenge for healthcare providers and public health programmes. In several sub‐Saharan African settings, soil consumption is embedded within cultural beliefs, traditional practices and perceptions of maternal well‐being [21, 39]. Therefore, interventions based solely on prohibition or risk messaging may have limited effectiveness. Instead, culturally sensitive approaches that engage community leaders, traditional birth attendants and other trusted stakeholders may be more successful in facilitating behavioural change while maintaining community acceptance [21, 22].

Evidence from maternal nutrition and health promotion programmes suggests that community engagement, nutrition education and improved access to micronutrient interventions may contribute to reducing behaviours associated with geophagy [11, 22]. Potential strategies include strengthening nutritional counselling during ANC visits, promoting dietary diversification, supporting iron–folic acid supplementation programmes and integrating geophagy screening into routine maternal health services [11, 14]. However, relatively few intervention studies have directly evaluated approaches specifically targeting geophagy during pregnancy. Consequently, further research is needed to identify effective, culturally appropriate and sustainable intervention models that can be integrated into existing maternal health programmes.

## 6. Research Gaps and Future Directions

Despite increasing recognition of geophagy as an important maternal health concern, substantial gaps remain in the evidence base. One major limitation is the predominance of cross‐sectional studies, which restrict the ability to establish temporal relationships or infer causality between geophagy and adverse maternal or pregnancy outcomes [16, 25, 54]. Longitudinal studies that follow women throughout pregnancy are needed to better understand patterns of geophagic behaviour, identify critical periods of exposure and clarify the extent to which geophagy contributes to nutritional deficiencies and adverse health outcomes independently of other risk factors.

Another important challenge is the lack of standardized approaches for measuring geophagy across studies and populations [12, 21]. Existing research employs diverse definitions and assessment methods, ranging from self‐reported frequency of soil consumption to estimates of quantity and type of geophagic material consumed. This methodological heterogeneity complicates comparisons across studies and limits the synthesis of evidence. The development of validated and culturally adaptable assessment tools would facilitate more reliable prevalence estimates, strengthen comparative research and support future meta‐analyses.

Future research should also evaluate the effectiveness of intervention strategies designed to address geophagy within maternal health programmes. Although nutritional counselling, micronutrient supplementation, dietary diversification and community‐based education are frequently recommended, evidence regarding their effectiveness in reducing geophagy remains limited [14, 45]. Integrating geophagy into broader maternal nutrition and antenatal care initiatives may provide a practical pathway for intervention; however, implementation strategies should be informed by robust evidence and adapted to local cultural contexts. Strengthening research in these areas will contribute to a more comprehensive understanding of geophagy and support the development of evidence‐based policies and programmes aimed at improving maternal and child health outcomes.

## 7. Limitations of the Review

This review has some limitations. As a narrative review, it did not follow the formal study selection and risk‐of‐bias assessment procedures used in systematic reviews. In addition, the evidence synthesized was largely derived from observational studies, limiting the ability to infer causal relationships between geophagy and reported maternal or pregnancy outcomes. Variations in study designs, populations and methods across the included studies may also have influenced the consistency and comparability of findings. Nevertheless, this review provides a comprehensive synthesis of the available evidence on geophagy during pregnancy in sub‐Saharan Africa and highlights key areas for future research.

## 8. Conclusion

Geophagy remains a common practice among pregnant women in many parts of sub‐Saharan Africa and is influenced by a complex interaction of cultural, nutritional, physiological and health‐system factors. Current evidence suggests that geophagy may adversely affect maternal nutritional status through reduced micronutrient bioavailability and increased exposure to toxicological and gastrointestinal risks. Although geophagy has been associated with adverse maternal and pregnancy outcomes, including anaemia, low birth weight and preterm delivery, much of the available evidence is observational, limiting conclusions regarding causality. Furthermore, evidence linking geophagy to NTDs remains limited and requires further investigation. Important research gaps persist, particularly regarding causal pathways, standardized methods for assessing geophagic practices and the effectiveness of intervention strategies. Given the cultural significance of geophagy in many communities, future efforts should combine rigorous research with culturally sensitive maternal nutrition and antenatal care interventions to better address this practice and its potential health implications.

## Funding

The authors acknowledge financial support from the Centre for Resilient Agri‐Food Systems (CRAFS) through the Regional Doctoral Scholarship Programme. The funding supports the corresponding author’s ongoing doctoral research.

## Conflicts of Interest

The authors declare no conflicts of interest.

## Data Availability

The authors have nothing to report.
